# Antibiogram Development in the Setting of a High Frequency of Multi-Drug Resistant Organisms at University Teaching Hospital, Lusaka, Zambia

**DOI:** 10.3390/antibiotics10070782

**Published:** 2021-06-28

**Authors:** Brenna M. Roth, Alexandra Laps, Kaunda Yamba, Emily L. Heil, J. Kristie Johnson, Kristen Stafford, Lottie M. Hachaambwa, Mox Kalumbi, Lloyd Mulenga, Devang M. Patel, Cassidy W. Claassen

**Affiliations:** 1 Center for International Health, Education, and Biosecurity, University of Maryland School of Medicine, Baltimore, MD 21201, USA; kstafford@ihv.umaryland.edu (K.S.); lhachaambwa@ihv.umaryland.edu (L.M.H.); cclaassen@ihv.umaryland.edu (C.W.C.); 2 Institute of Human Virology, University of Maryland School of Medicine, Baltimore, MD 21201, USA; dpatel@ihv.umaryland.edu; 3 University of Maryland School of Medicine, Baltimore, MD 21201, USA; alaps831@gmail.com; 4 Department of Pathology and Microbiology, University Teaching Hospital, Lusaka 10101, Zambia; kaundayamba@gmail.com (K.Y.); moxmalamakalumbi@gmail.com (M.K.); 5 Department of Pharmacy Practice and Science, University of Maryland School of Pharmacy, Baltimore, MD 21201, USA; eheil@rx.umaryland.edu; 6 Department of Pathology, University of Maryland School of Medicine, Baltimore, MD 21201, USA; jkjohnson@som.umaryland.edu; 7 Department of Epidemiology and Public Health, University of Maryland School of Medicine, Baltimore, MD 21201, USA; 8 Department of Medicine, University of Zambia School of Medicine, Lusaka 10101, Zambia; lbmulenga@yahoo.com; 9 Infectious Diseases Unit, Department of Internal Medicine, University Teaching Hospital, Lusaka 10101, Zambia; 10 Ministry of Health, Government of the Republic of Zambia, Lusaka 10101, Zambia

**Keywords:** bacteria, antimicrobial resistance, multi-drug resistant organisms, antibiogram, antimicrobial stewardship, Zambia

## Abstract

Antimicrobial resistance is a global challenge requiring reliable surveillance data collection and use. Prior studies on resistance in Zambia depended on laboratory methods with limited standardization. Since 2015, the University Teaching Hospital (UTH) microbiology laboratory has used the Vitek 2 Compact (bioMerieux, Inc., Marcy-l’Étoile, France) for standardized identification and susceptibility testing. We conducted a cross-sectional study of 2019 bacterial isolates collected from July 2015 to April 2017 to identify bacterial causes of infections, their susceptibility to commonly used antibiotics at UTH, and develop hospital antibiograms with a multidisciplinary team using World Health Organization guidance. We found high levels of antibiotic resistance among Gram negative bacteria. *Escherichia coli* and *Klebsiella pneumoniae* were highly resistant to all antibiotics except amikacin and carbapenems. *E. coli* had susceptibilities of 42.4% to amoxicillin/clavulanic acid, 41.4% to ceftriaxone, 40.2% to ciprofloxacin, and 10.4% to trimethoprim/sulfamethoxazole (TMP/SMX). *K. pneumoniae* had susceptibilities of 20.7% to amoxicillin/clavulanic acid, 15.6% to ceftriaxone, 48.5% to ciprofloxacin, and 12.3% to TMP/SMX. The high resistance to 3rd generation cephalosporins indicates high rates of beta-lactamase production. This is information that clinicians need to inform clinical decision making and choice of empiric antibiotics and that UTH requires to inform antimicrobial stewardship such as improvements in antibiotic use.

## 1. Introduction

Antimicrobial resistance (AMR) is a worldwide public health crisis with potentially severe implications for resource-limited settings. Data on AMR in sub-Saharan Africa (SSA) is sparse, but national data reported by the World Health Organization (WHO) indicates *Escherichia coli* and *Klebsiella pneumoniae* resistance to 3^rd^ generation cephalosporins at 2–70% and 8–77%, respectively [1]. *E. coli* resistance to fluoroquinolones is reported at 14–71% [1]. Methicillin-resistant *Staphylococcus aureus* (MRSA) is reported to make up 12–80% of *S. aureus* isolates while *Streptococcus pneumoniae* resistance to penicillin is at 3–16% [1].

Available literature from SSA supports this national data. *E. coli* and *K. pneumoniae* generally have low susceptibility to penicillin, cephalosporins, fluoroquinolones, and trimethoprim/sulfamethoxazole (TMP/SMX) while maintaining high susceptibility to carbapenems and amikacin [2,3,4,5,6,7,8,9]. The frequency of MRSA among *S. aureus* ranges from 2.2% to 31.3% [2,8,10,11]. Data on *S. pneumoniae* is often grouped with other *Streptococcus* species and shows high resistance to penicillin, up to 30.4% [6,12,13]. *Enterococcus* is often not reported as *Enterococcus faecium* and *E. faecalis* giving an unclear picture of *E. faecium* resistance to ampicillin [6,12].

The existing literature on AMR in Zambia is limited. One study that evaluated neonatal sepsis found resistance of *Klebsiella* species and *E. coli* at 71–100% and 50–100% for all drugs except imipenem (0–1% resistant) [14]. A study that evaluated *K. pneumoniae* as a cause of neonatal sepsis found resistance of 95.5–100% to all antibiotics except amikacin (0%) and carbapenems (0%) [15]. Another study that evaluated causes of diarrhea among children found that 66.7% of *E. coli* were extended-spectrum beta lactamase (ESBL)-producers [16]. A study that included adults found less resistance of *E. coli* and *Klebsiella* species to fluoroquinolones, 0–24.1% and 0%, respectively, although the number of specimens tested was as low as one [17]. Another study found 43% of *S. aureus* isolates were MRSA with 100% resistance to TMP/SMX [18]. A study that looked at *E.coli* as a cause of gastroenteritis in people infected with HIV and in people not infected with HIV found high resistance to TMP/SMX and nalidixic acid [19]. The broad applicability of these studies is limited, but they support the important role of microbiology laboratories in Zambia to conduct resistance testing which can then guide clinical care [20,21].

Previously, University Teaching Hospital (UTH) developed an antibiogram based on data collected from 2012–2013 and the 2012 Clinical and Laboratory Standards Institute (CLSI) guidelines. Individual antibiograms were developed by specimen type and for the neonatal ICU. *E. coli* was 39–89% resistant to cefotaxime and 38–71% resistant to fluoroquinolones (internal data, unpublished). *K. pneumoniae* was 49–100% resistant to cefotaxime and 40–95% resistant to fluoroquinolones (internal data, unpublished). *Enterococcus* was not reported. Based on susceptibility to cefoxitin and oxacillin, MRSA comprised 57–67% of *S. aureus* specimens. *S. pneumoniae* did not have susceptibility to penicillin reported for some specimens but was 25% resistant for other specimens while maintaining susceptibility to ceftriaxone (internal data, unpublished).

The WHO emphasizes the key role of the microbiology laboratory in antimicrobial stewardship (AMS) by informing the appropriate use of antibiotics through development of antibiograms [22]. In 2017, the UTH microbiology lab instituted the use of Vitek 2 Compact (bioMerieux, Inc., Marcy-l’Étoile, France), which offers reliable and standardized identification and susceptibility testing of bacteria and yeast [23,24,25] as quality control is maintained at UTH. The purpose of this study was to: 1. characterize patterns of AMR at UTH based on susceptibility data from Vitek 2 Compact, and 2. use these results to develop an antibiogram to inform AMS efforts at UTH and guide the empiric use of antibiotics.

## 2. Results

### 2.1. Gram-Negative Organisms

**Figure 1:**
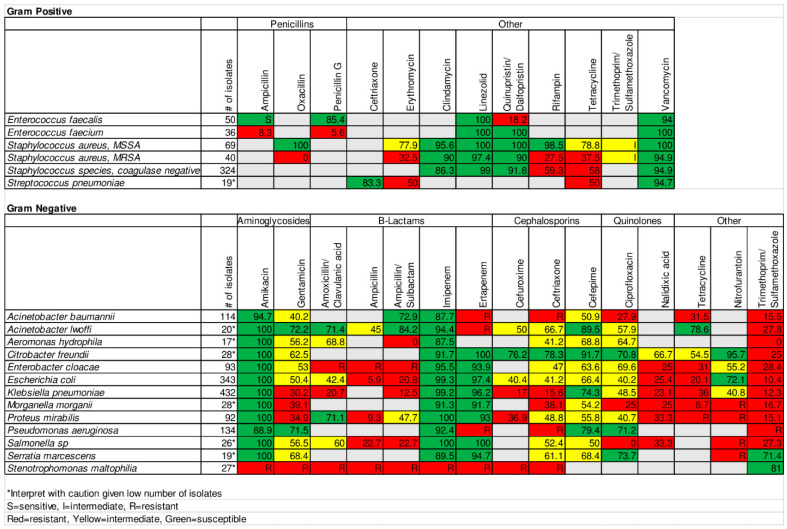
UTH antibiogram for infectious disease specialists.

*E. coli* and *K. pneumoniae* were the most isolated Gram negative (GN) bacteria, with 343 (25%) and 432 (31.5%) specimens, respectively. They were highly resistant to all antibiotics except amikacin and carbapenems (Figure 1). *E. coli* had susceptibilities of 42.4% to amoxicillin/clavulanic acid, 41.4% to ceftriaxone, 40.2% to ciprofloxacin, and 10.4% to TMP/SMX. *K. pneumoniae* had susceptibilities of 20.7% to amoxicillin/clavulanic acid, 15.6% to ceftriaxone, 48.5% to ciprofloxacin, and 12.3% to TMP/SMX. The high resistance to 3^rd^ generation cephalosporins indicates high rates of beta-lactamase production including ESBLs and AmpC beta lactamases [26].

### 2.2. Gram-Positive Organisms

There were 324 (60.2%) Staphylococcus species, coagulase negative (CoNS) isolates, 109 (20.3%) *S. aureus* isolates, 86 (16%) Enterococcus isolates, and 19 (3.5%) *S. pneumoniae* isolates (Figure 1). CoNS had decreased susceptibility to tetracycline (58%), while maintaining susceptibility to vancomycin, linezolid, and quinupristin/dalfopristin. MRSA made up 37% of *S. aureus* isolates. MRSA and methicillin susceptible *S. aureus* (MSSA) had very low susceptibility (7.5% and 27.9%, respectively) to TMP/SMX. MSSA had high susceptibility to other drugs against which it was tested. MRSA had decreased susceptibility to tetracycline (38%) and erythromycin (33%), while maintaining susceptibility to vancomycin, linezolid, and quinupristin/dalfopristin. *S. pneumoniae* was 83.3% sensitive to ceftriaxone. *E. faecalis* susceptibility to ampicillin was only 89%. This susceptibility was lower than expected and we presumed it was an error and chose to label it as sensitive and not report the percentage in the infectious disease (ID) antibiogram to avoid leading providers to underutilize ampicillin. *E. faecium* was 8.3% sensitive to ampicillin. Both maintained susceptibility to vancomycin.

## 3. Discussion

In this study, we analyzed patient isolates from UTH and found high levels of resistance, particularly among GN bacteria. While we could not test for broad-spectrum beta-lactamase production, the high rates of resistance to 3^rd^ generation cephalosporins, 41.4% susceptibility of *E. coli* and 15.6% susceptibility of *K. pneumomiae* to ceftriaxone, respectively, indicate this is common. This is not entirely unexpected as 3^rd^ generation cephalosporins are the most commonly utilized antibiotics at UTH, driving the development of ESBL-producing organisms [27]. Studies from SSA reveal high rates of ESBL production [16,28] or resistance to 3^rd^ generation cephalosporins [2,3,4,5,6,7,8,9]. A study from Malawi saw an increase in ESBL-production from 2003 to 2016 in *E. coli* from 0.7% to 30.3% and in *Klebsiella* species from 11.8% to 90.5% [12]. We also found high levels of resistance to fluoroquinolones, 40.2% susceptibility of *E. coli* and 48.5% susceptibility of *K. pneumoniae* to ciprofloxacin, respectively, consistent with findings from other studies [2,3,5,7,9]. The study from Malawi found resistance to ciprofloxacin rose from 2.5% to 31.1% in *E. coli* and 1.7% to 70.2% in *Klebsiella* species between 2003 and 2016 [12].

Our findings of high resistance to TMP/SMX are consistent with other studies and may result from the use of TMP/SMX for prevention of opportunistic infections in people living with HIV [2,3,4,5,6,9,14,15] and/or overuse of antibiotics generally. These findings are concerning because TMP/SMX is commonly used for empiric treatment, particularly skin and soft tissue infections.

The large number of CoNS isolates may indicate high levels of specimen contamination. CoNS is often not clinically significant; however, we were not able to ascertain the clinical significance of these specimens with the data gathered and analyzed for our study.

MRSA made up a higher proportion (37%) of *S. aureus* isolates at UTH compared to that reported in much of the literature in SSA, 2.2% to 45% [2,3,7,9,10,11,12,18]. MSSA was susceptible to a broader range of antibiotics compared to MRSA, emphasizing the importance of reporting the sensitivities of MSSA and MRSA separately. These findings are important because of the limited availability of intravenous vancomycin and other antibiotics that cover serious systemic MRSA infections.

*S. pneumoniae* isolates at UTH were highly susceptible to ceftriaxone. The literature from SSA contains less information on susceptibility to ceftriaxone and cefuroxime compared to penicillin. Penicillin susceptibility was not reported at UTH. A survey that examined data from the Democratic Republic of Congo, Ivory Coast, Kenya, and Senegal revealed a range from 69.6% to 100% susceptibility to penicillin and 96.4% susceptibility to ceftriaxone for Kenya [13]. A study from Ghana found 32.5% susceptibility to penicillin and 69.2% susceptibility to cefuroxime [6].

*E. faecium* showed high resistance to ampicillin and penicillin. It is difficult to compare this to findings from studies from SSA as they report *Enterococcus* species rather than individual species. Studies from Ghana [6] and Malawi [12] showed high resistance to ampicillin, likely due to the presence of *E. faecium*. A study from Rwanda, however, found high susceptibility of *E. faecium* to ampicillin [2]. Separating *E. faecalis* from *E. faecium* allows a broader range of antibiotics to be available for *E. faecalis*.

These findings indicate that a carbapenem is the most appropriate empiric antibiotic in patients with suspected GN infections at UTH, especially those caused by *E. coli* and *K. pneumoniae*. This is a concerning finding that emphasizes the need for a robust AMS program that can implement interventions to improve antibiotic use and preserve the availability of antibiotics. UTH is currently developing its AMS program and while ID specialists are available, they are not consulted on all cases of infection and there is no other oversight or feedback to clinicians to advise on appropriate antibiotic use.

AMS interventions improve antibiotic use through education, audits of antibiotics, and feedback to providers [29]. There is little literature from SSA on AMS interventions; however, one study from South Africa revealed that AMS can be implemented in that context and have significant impacts in decreasing days of antibiotic consumption [30]. While antibiograms are an important tool in AMS with their use recommended by WHO [22], the technical expertise required for their development is often not present in facilities and contributes to challenges in their preparation [31].

**Figure 2:**
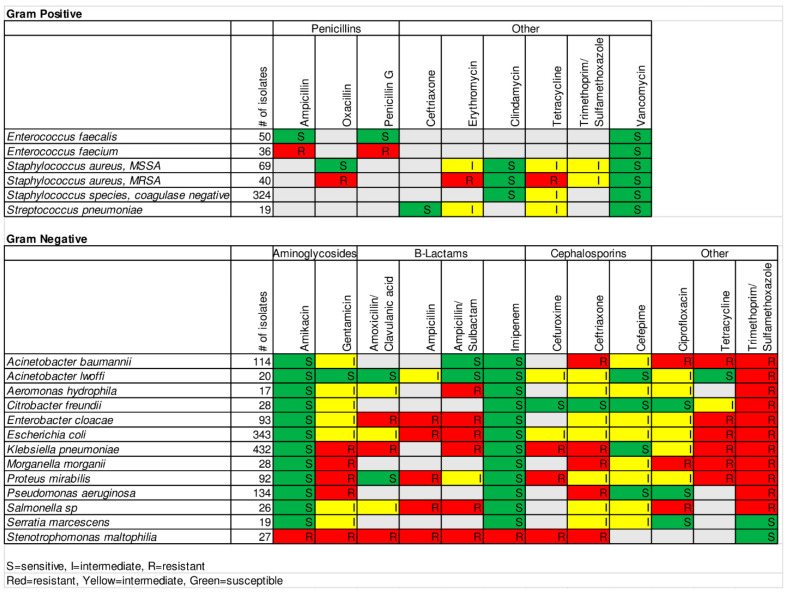
UTH antibiogram for general practitioners.

A strength of our project was the unique expertise available at UTH as a result of, among other things, a robust training program for ID physicians [32,33] and strong collaboration among ID physicians, ID pharmacists, and microbiologists at UTH and the University of Maryland Baltimore (UMB). This interdisciplinary group has also collaborated to study antibiotic prescribing patterns at UTH. This relatively unique expertise in SSA allowed us to develop antibiograms to meet the needs of ID specialists (Figure 1) and general practitioners (Figure 2) at UTH.

For the general practitioner antibiogram, we took into consideration the high resistance of GN organisms to most antibiotics. We decided to set the susceptibility threshold for GN organisms to maintain a wider range of options for empiric therapy, providing options other than carbapenems. This change in threshold and the desire to simplify use of the antibiogram by avoiding interpretation of percentages by non-specialist practitioners led us to recognize the need to develop two versions of the antibiogram. We wanted to ensure ID specialists, who have the expertise to interpret the more detailed information, had access to this information for antibiotic selection in complex cases.

Another strength of this study is the consistency that automated Vitek 2 Compact susceptibility testing offered compared to technician dependent manual methods that require training to maintain consistency. To our knowledge, this is one of the first such studies in SSA. While we recognize that Vitek 2 has limitations in accuracy [34,35,36,37], quality control of bench procedures at UTH is more variable than that of Vitek 2 Compact, performed quarterly, making these methods less consistent.

Many studies from SSA do not report the use of standard interpretation guidelines such as those of CLSI or European Committee on Antimicrobial Susceptibility Testing (EUCAST) that affects interpretation of results. We used CLSI 2015 guidelines, in use at the laboratory at UTH at the time of specimen collection, to determine MICs used for interpretation and develop the antibiogram. Moving forward, updated antibiograms at UTH should utilize the most up-to-date versions of the CLSI or EUCAST guidelines.

A significant limitation of this study is limited isolates for certain bacteria. WHO recommends a minimum of 30 isolates to maintain statistical validity [38]. The 30 isolates come from the most recent one-year period; however, the WHO recommends adding the year previous to that, if 30 isolates are not reached. Our dataset starts when Vitek 2 Compact was initiated at UTH, went to the time of data collection, for a total of 22 months of data. Despite this, some organisms had as few as 17 isolates. The development of future antibiograms at UTH may require data from a longer time. Alternatively, there may need to be improvement in the quality or quantity of cultures. Moreover, incomplete identifying information may have allowed duplicates to remain in the data presented, affecting the results. We estimate the effect to be small as the number of possible duplicates was low. Last, it is difficult to know the true percentage of ESBL-producing *K. pneumoniae* and *E. coli* because UTH does not routinely test for ESBLs. Due to limitations in materials for cultures (e.g., blood culture bottles), routine culturing is not done for all patients with suspected infections. Therefore, the findings in the antibiogram may be biased towards sicker patients with potentially more resistant organisms than if culture practices were more widespread.

An additional limitation is that we were unable to differentiate between all specimens obtained from inpatient and outpatient settings. We were, thus, unable to develop separate antibiograms for each setting as the fidelity of location information was questionable and sample sizes would have decreased. Overcoming these limitations in the future will further improve empiric antibiotic therapy in both settings as different clinical settings and patient populations can have varying patterns of resistance.

## 4. Materials and Methods

### 4.1. Patients and Study Site

This was a cross-sectional study undertaken at UTH in Lusaka, Zambia. UTH is a 1655-bed teaching hospital that trains medical students, nurses, and pharmacists and serves as a referral hospital for the country. UTH trains Zambian physicians in advanced HIV medicine and infectious diseases through a collaboration between the Ministry of Health, UTH, the University of Zambia, and UMB [32,33].

We collected all data from bacterial isolates from July 2015 to April 2017 with susceptibility testing performed using Vitek 2 Compact by the microbiology laboratory from the inpatient and outpatient settings, both pediatrics and adults.

### 4.2. Lab Techniques

Specimens from blood, cerebrospinal fluid, body fluid, sputum, urine, and wounds were collected, processed, and analyzed in the microbiology laboratory per UTH guidelines. Growth on culture of bacterial isolates that required susceptibility testing were run on Vitek 2 Compact. Identification of organisms was made using Vitek 2 GP, Vitek 2 GN, Vitek 2 Streptococcus, Vitek 2 YST, Vitek 2 NH, and Vitek 2 CBC cards. Susceptibility testing was performed using the Vitek AST-GP67, Vitek AST-GN86, or Vitek AST-01 cards following CLSI guidelines [21]. The lab did not test for ESBLs and AmpC beta lactamases using Vitek 2 Compact.

### 4.3. Data Collection

We collected data from all isolates, 2019, run on Vitek 2 Compact from the start of its use at UTH in July 2015 through April 2017 when data collection occurred. The data was entered into WHONET 5.6, a free Windows-based database software developed for the management of microbiology laboratory data. The data entered for each culture specimen included specimen number, sex, date of birth, age category (newborn, pediatric, adult), department, location (inpatient, outpatient), specimen date, reason for culture, and organism. For each specimen, we entered the minimum inhibitory concentrations (MICs) into WHONET, which determined the percentage of each organism susceptible to each drug against which it was tested.

### 4.4. Data Analysis

WHONET aggregated and analyzed the data. If there was more than one isolate of the same organism for the same patient during the same admission, we kept the first isolate in the dataset independent of the resistance of the isolates. Missing data elements sometimes made it difficult to confirm isolates from the same patient. If unclear, isolates remained in the dataset. Vitek adds the MICs for TMP and SMX together but uses only the MIC for TMP to set the breakpoint. This error carries over into WHONET. We corrected the TMP/SMX breakpoints in the WHONET aggregated dataset.

### 4.5. Antibiogram Development

Aggregated data from WHONET produced susceptibility percentages for every organism. A team that included physicians, pharmacists, and microbiologists from the UTH in Lusaka and UMB reviewed these auto-generated susceptibilities. We initially excluded organisms not commonly associated with disease or with fewer than 30 isolates, given the potential for diminished accuracy [22]. We then reviewed the list and chose to include clinically important organisms despite having fewer than 30 isolates with the notation that these results should be interpreted with caution based on the low number of isolates. The antibiotics included in the antibiogram were narrowed to those commonly available at UTH.

We developed one antibiogram for ID doctors with specific percentage details for a wide range of antibiotics. We developed a second antibiogram for general practitioners with fewer antibiotic options and summarized antibiotic sensitivity as resistant (“R”), intermediate (“I”), and sensitive (“S”) to communicate when an antibiotic should be used to treat a particular bacteria. A common, but not universal, practice is to define susceptible as 80% to 100% susceptible, intermediate as 60% to 79.9% susceptible, and resistant as 0–59.9% susceptible. We used these ranges for GP organisms. The high resistance in GN organisms led us to lower the threshold for susceptible and intermediate to give practitioners a wider range of antibiotic options. Thus, susceptible was defined as 70% to 100% susceptible, intermediate as 40% to 69.9% susceptible, and resistant as 0–39.9% susceptible. In the instance of intrinsic resistance of an organism to an antibiotic, this was labeled as “R” rather than providing the percentage susceptible. A panel of ID doctors, an ID pharmacist, and a microbiologist reviewed the antibiograms. This review led to further refinement of the antibiograms, for example, in the case of *E. faecalis* and ampicillin as explained above.

### 4.6. Ethics Statement

This study was approved by the University of Zambia Biomedical Research Ethics Committee Institutional Review Board (IRB) (reference number 009-06-17) and the UMB IRB (study number HP-00076126). This was a retrospective study of de-identified specimens, so IRBs waived the requirement for informed consent.

## 5. Conclusions

Improper and overuse of antibiotics is a multifaceted problem that is contributing to global AMR. We identified high rates of AMR at UTH, a tertiary teaching and referral hospital in Zambia. The high degrees of resistance in GN and GP organisms found in our study highlight the need for reliable microbiology laboratory data that can be used to consistently and regularly develop, update, and disseminate antibiograms to inform the appropriate use of antibiotics and other AMS efforts at UTH.

Antibiograms provide guidance on the effective management of infections and the appropriate use of empiric antibiotic therapy based on local AMR patterns. We utilized consistent susceptibility data from Vitek 2 Compact along with the expertise of ID physicians, ID pharmacists, and microbiologists from UTH and UMB to develop antibiograms for ID and general physicians at UTH to enhance their ability to choose antibiotics and provide UTH with critical information to inform AMS efforts.

Future efforts should include educating providers on the use of antibiograms, developing guidelines for clinicians on the appropriate use of antibiotics, and focusing quality improvement efforts on particular infections or antibiotics which has been proven effective in other settings [39,40]. It is also essential to build the capacity of microbiology laboratories to improve the quality and validity of all microbiology testing to ensure that the data in the antibiograms is reliable. Further, research is needed to assess the impact of deploying antibiograms and antibiotic guidelines at UTH.

## Data Availability

The data belongs to the University Teaching Hospital of Zambia and are not publicly available. Data are available from the authors upon reasonable request and with permission from the University Teaching Hospital Microbiology Laboratory, Lusaka, Zambia.
